# An unusual case of invasive rhino-cerebral aspergillosis in a pediatric patient: A case report with diagnostic approach and imaging review

**DOI:** 10.1016/j.radcr.2026.02.053

**Published:** 2026-03-21

**Authors:** Amal Akammar, Zaid EN Nasery, Djoudline Doughmi, Hajar Ouazzani Chahdi, Ismail Chaouch, Nizar El Bouardi, Badreeddine Alami, Moulay Youssef Alaoui Lamrani, Mustapha Harandou, Mustapha Maaroufi, Meriem Boubbou

**Affiliations:** a Mother and Child Radiology Department, Hassan II University Hospital Fez, Sidi Mohamed Ben Abdellah University, Fez, Morocco; b Anesthesi Department, Hassan II University Hospital Fez, Sidi Mohamed ben Abdellah University, Fez, Morocco; c Department of Central Radiology, Hassan II University Hospital Fez, Sidi Mohamed ben Abdellah University, Fez, Morocco

**Keywords:** Aspergillosis, Pediatric, Rhino-cerebral infection, Imaging

## Abstract

Cerebral aspergillosis is a rare and often fatal fungal infection, particularly in immunocompromised individuals. Diagnosis is challenging due to nonspecific clinical and radiological findings. We report the case of a 14-year-old girl with a history of type 1 diabetes who presented with severe diabetic ketoacidosis, rapidly followed by altered consciousness and a necrotic nasal lesion. Radiological investigations revealed multifocal hemorrhagic infarcts and abscesses, and a biopsy confirmed invasive rhino-cerebral aspergillosis due to *Aspergillus fumigatus*. Despite prompt antifungal therapy with voriconazole, the patient succumbed to the disease. This case underscores the aggressive nature of this infection in diabetic patients and emphasizes the critical role of early neuroimaging when clinical suspicion arises.

## Introduction

*Aspergillus* is a ubiquitous fungus and is the most common fungal pathogen in sinus disease. Cerebral aspergillosis presents significant diagnostic challenges and predominantly occurs in immunocompromised individuals [[1], [2], [3]]. The central nervous system (CNS) can become involved either through hematogenous dissemination from an extracranial focus, typically the lungs, or via direct extension from the paranasal sinuses [4].

Despite advances in medical imaging and antifungal therapies, the prognosis of cerebral aspergillosis remains poor, with mortality rates approaching 100% in some cases [5]. Early and accurate diagnosis is critical for initiating appropriate treatment, yet the nonspecific clinical and radiological findings often delay this process. The incidence of invasive fungal infections (IFIs) may be rising in the context of post-critical illness states and poorly controlled metabolic conditions like diabetes, making awareness of this entity increasingly relevant [5].

## Case presentation

A 14-year-old girl with a history of type 1 diabetes was admitted to the pediatric intensive care unit (PICU) for the management of severe diabetic ketoacidosis. Upon admission, the patient presented with altered consciousness (Glasgow Coma Scale score of 8). Initial management included intravenous insulin therapy and fluid resuscitation. However, her condition rapidly deteriorated with signs of circulatory collapse, requiring endotracheal intubation and hemodynamic support with norepinephrine. She developed acute kidney injury with anuria and hyperkalemia, complicated by acute pulmonary edema. This necessitated treatment with intravenous furosemide, followed by the initiation of renal replacement therapy (hemodialysis) due to refractory fluid overload and metabolic derangements. Broad-spectrum empiric antibiotic therapy was initiated with amoxicillin–clavulanate, ciprofloxacin, and metronidazole.

*Between Day 5 and Day 10,* there was no clinical improvement despite intensive management. A brain CT scan performed returned normal. Subsequently, the patient developed a nosocomial pulmonary infection characterized by clinical deterioration and a marked inflammatory syndrome, with an elevated white blood cell count and C-reactive protein levels.

**Fig. 1:**
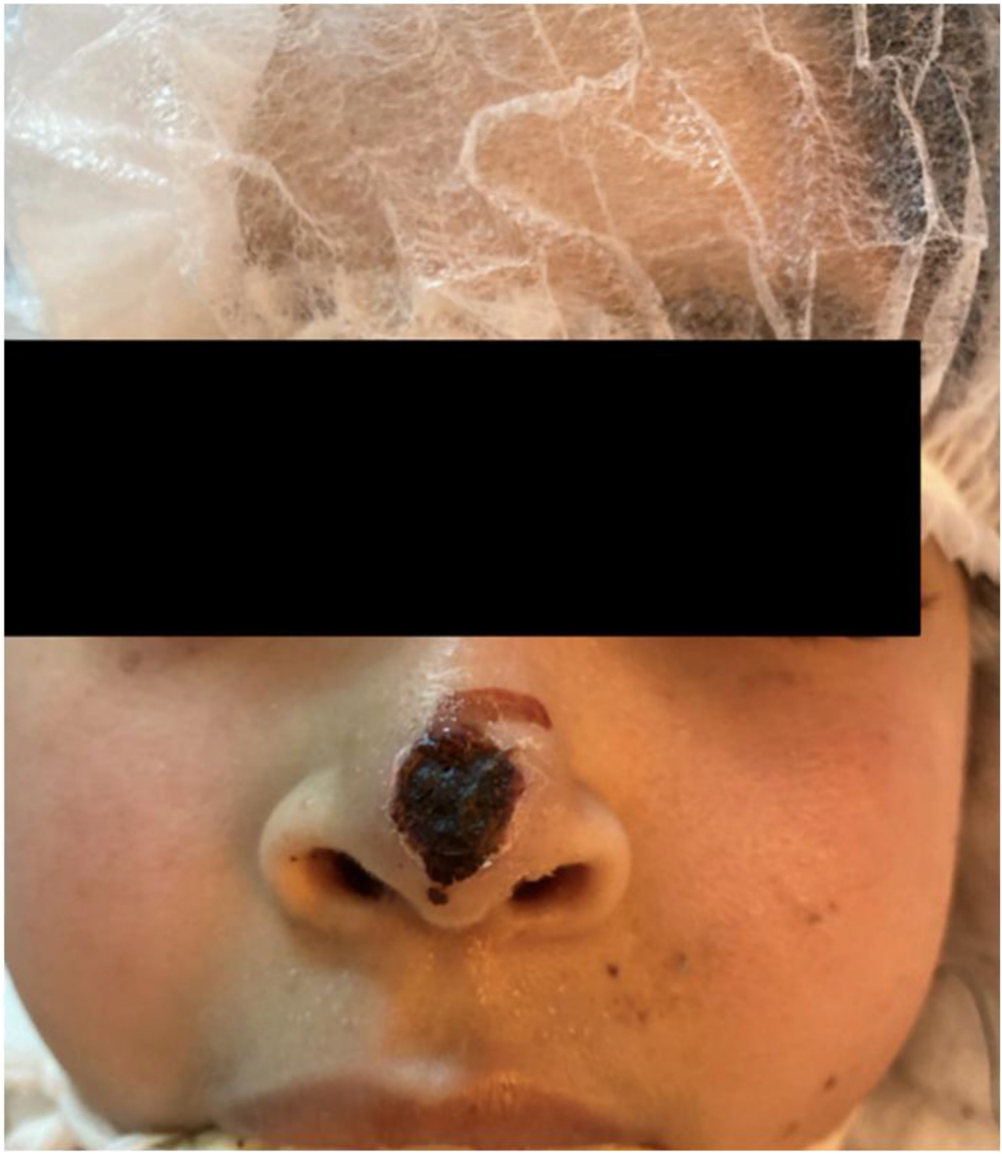
Clinical photograph of nasal necrosis.

*Diagnostic turning point (Day 11):* A clinical turning point occurred with the appearance of a necrotic macule on the nasal septum, raising suspicion of an IFI (Fig. 1). Antibiotic therapy was escalated to tigecycline. A "sedation holiday" was attempted to assess neurological status; the patient exhibited spontaneous eye-opening and limited contact but remained severely impaired. A biopsy of the nasal lesion was performed immediately.

**Fig. 2:**
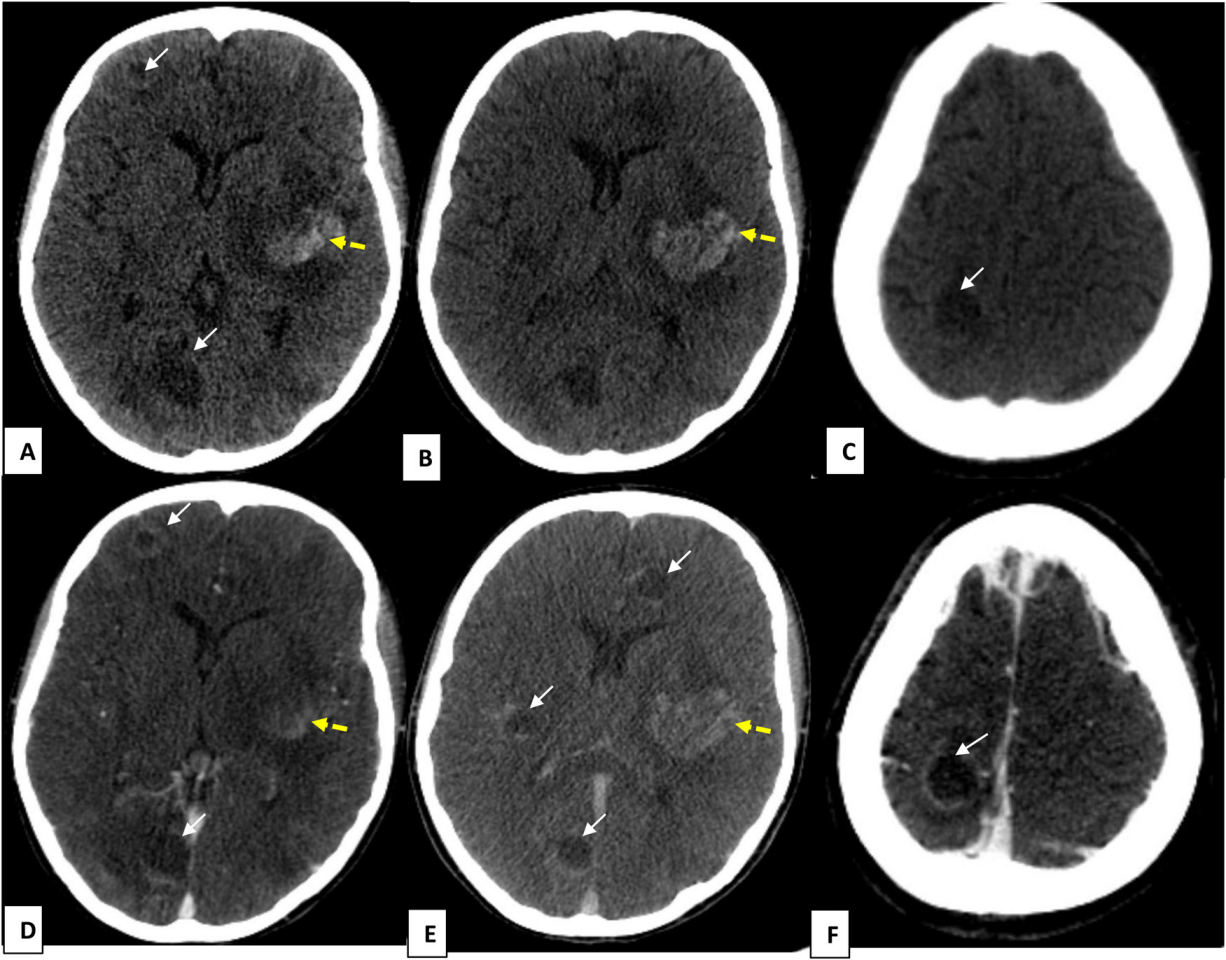
Brain CT scan before (A–C) and after contrast injection (D–F) shows multiple supratentorial cerebral lesions with fluid-filled centers and smoothly enhancing walls after contrast administration (white arrows), associated with a left capsulolenticular hematoma (yellow-dashed arrows), with associated surrounding edema.

**Fig. 3:**
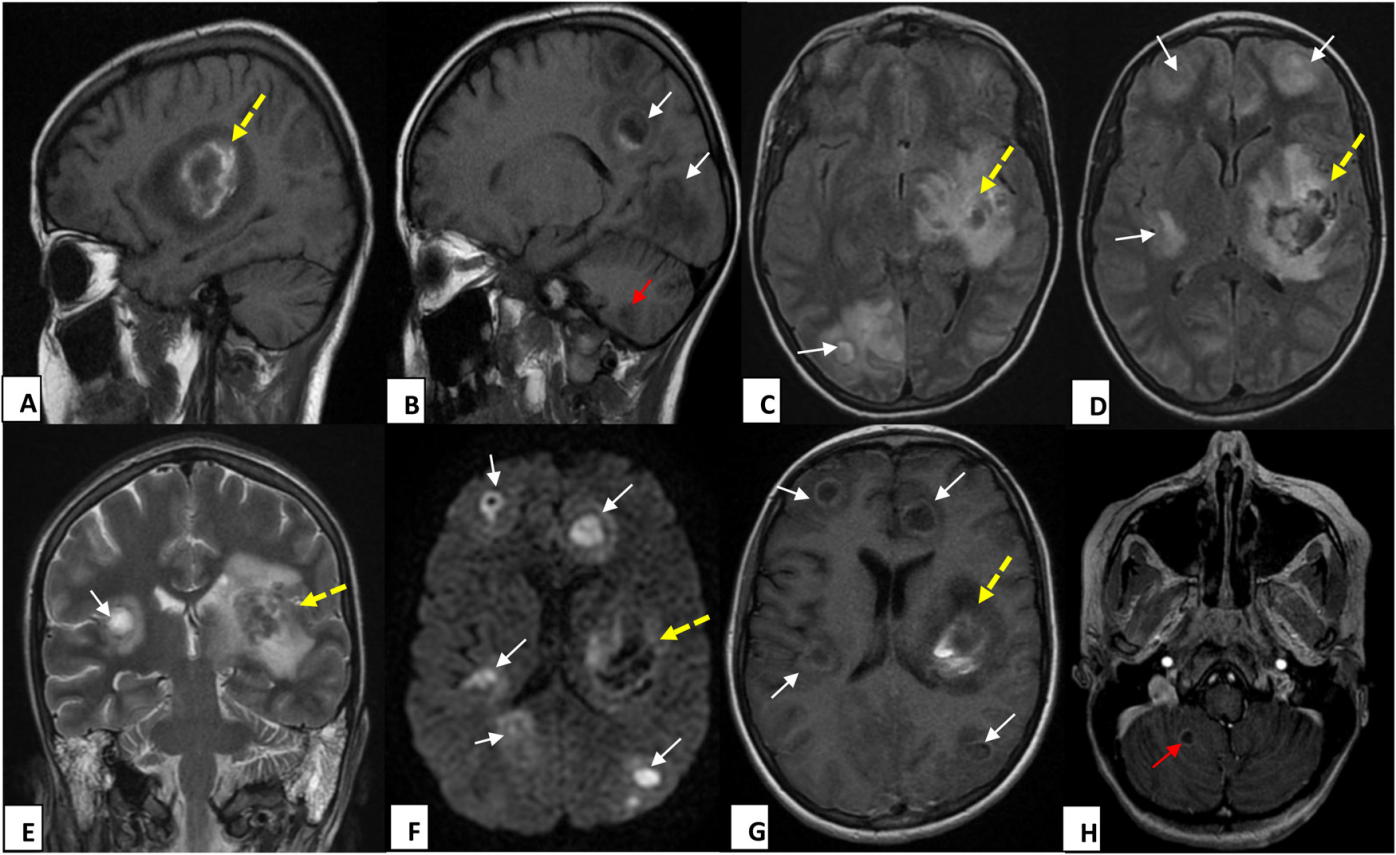
Brain MRI reveals a large, heterogeneous left capsulo-lenticular hematoma (yellow dashed arrows), characterized by peripheral hyperintensity on T1-weighted images (A, B) and significant perilesional edema on FLAIR (C, D) and T2-weighted sequences (E), causing a discrete mass effect. Concurrently, multiple cystic lesions that are hyperintense on T2/FLAIR, with hemorrhagic components on T1-weighted sequences and ring-like enhancement after contrast administration with ring-enhancing abscesses with irregular thick walls, are identified bilaterally at the gray–white matter junction (white arrows) and in the right cerebellar hemisphere (red arrows in B and H). These disseminated lesions exhibit restricted diffusion on DWI (F), consistent with septic emboli and purulent content.

*On Day 15*, due to persistent neurological impairment and the evolving nasal lesion, a second brain CT was performed. It revealed a left capsulolenticular intraparenchymal hematoma alongside multiple small bilateral intra-axial lesions at the gray–white matter junction, suggestive of a disseminated fungal infection (Fig. 2). Subsequent Magnetic Resonance Imaging (MRI) provided further characterization (Fig. 3), confirming a large, heterogeneous hemorrhagic lesion in the left capsulolenticular region with associated mass effect and vasogenic edema. Additionally, multiple small, ring-enhancing abscesses were identified bilaterally at the gray–white matter junction and in the cerebellum. These lesions exhibited restricted diffusion on diffusion-weighted imaging (DWI) sequences, characteristic of septic emboli and fungal abscesses.

Although the imaging findings were highly suggestive, several differential diagnoses were considered, including *mucormycosis* and *bacterial abscesses*, which can present similarly in pediatric diabetic patients. Distinguishing features were carefully evaluated: Unlike bacterial abscesses, which typically show a uniform, thin, and smooth ring enhancement, these fungal lesions displayed thicker, irregular walls with internal projections. Furthermore, although both *Aspergillus* and *Mucorales* are angioinvasive, mucormycosis often presents with even more aggressive cavernous sinus involvement and a higher propensity for rapid bone destruction. In this case, the specific pattern of multiple small lesions at the gray–white matter junction, combined with the restricted diffusion on DWI, strongly pointed toward septic fungal emboli. The definitive diagnosis was ultimately provided by the nasal biopsy, which identified *Aspergillus fumigatus* and ruled out the non-septate hyphae characteristic of mucormycosis.

Following clinical suspicion and subsequent confirmation, targeted antifungal therapy with *voriconazole* was initiated. A neurosurgical consultation was requested; however, given the multifocality and extensive nature of the cerebral lesions, the patient was deemed ineligible for surgical intervention. Despite the addition of antifungal treatment and maximal supportive care, the patient’s condition continued to decline due to the opportunistic infection and severe cerebral edema. She suffered a cardiac arrest on Day 19 and passed away.

## Discussion

Cerebral aspergillosis remains one of the most devastating opportunistic infections; while historically confined to severely immunocompromised populations, the epidemiological landscape has undergone a paradigm shift. Recent data (2024-2025) indicate a *5% annual increase* in prevalence, a trend significantly accelerated by the rise of secondary immunodeficiencies in the post-COVID-19 era. Currently, there is growing recognition of IFIs in "non-traditional" hosts, particularly those with metabolic dysregulation. In uncontrolled diabetic states, the combination of hyperglycemia and acidosis serves as a dual catalyst: It provides an ideal substrate for fungal proliferation while simultaneously impairing neutrophil chemotaxis and phagocytosis [1]. Beyond its shifting epidemiology, the disease presents significant diagnostic challenges. Its clinical presentation is often nonspecific, and varied imaging findings frequently mimic other intracranial pathologies, such as primary tumors or bacterial abscesses. This diagnostic mimicry, combined with the fungus's angioinvasive nature, necessitates a multidisciplinary approach for timely identification [1].

The pathogenesis of cerebral aspergillosis involves the angioinvasive nature of *Aspergillus* species, which leads to vascular thrombosis, infarction, and hemorrhage. The fungus typically enters the central nervous system through hematogenous spread or by direct extension from the paranasal sinuses. The preferential involvement of the gray–white matter junction and deep gray nuclei on imaging is a direct result of this vascular tropism. These areas are supplied by small, perforating end-arteries, which are susceptible to occlusion by fungal emboli. This explains the radiological phenotype of multifocal, often hemorrhagic, lesions observed in our patient [[6], [7], [8]].

CT scans are often the first imaging modality used in patients presenting with neurological symptoms. CT can reveal hypodense lesions indicative of infarcts, which are caused by the occlusion of cerebral vessels by *Aspergillus* hyphae. These infarcts often occur at the gray–white matter junction, a common site for embolic events, and may be associated with hemorrhage, leading to mixed-density lesions. The presence of ring-enhancing lesions on contrast-enhanced CT scans is suggestive of abscess formation, a late-stage complication of cerebral aspergillosis [9]. In our case, CT imaging revealed multiple hypodense areas consistent with infarction, as well as areas of hemorrhage, confirming the angio-invasive nature of the infection.

MRI is the gold standard for diagnosing cerebral aspergillosis due to its superior soft tissue contrast and multiplanar imaging capabilities. It is particularly effective in detecting early ischemic changes, inflammation, and abscess formation. T1-weighted sequences typically show hyperintensity lesions, reflecting necrosis or hemorrhage, while T2-weighted and FLAIR sequences often reveal hyperintense lesions indicative of edema and infarction. The involvement of the gray-white matter junction, basal ganglia, and thalami on MRI is characteristic of *Aspergillus* infection due to the predilection of the fungus for small perforating arteries in these regions [[6], [7], [8]].

One of the hallmark findings in cerebral aspergillosis on MRI is the presence of ring-enhancing lesions, indicative of abscess formation. These lesions typically exhibit a hypointense rim on T2-weighted images, which may be due to the presence of hemorrhage, fungal elements, or iron deposition from hemoglobin breakdown. The irregular and thickened appearance of these rims, compared to the more uniform appearance seen in bacterial abscesses, is a distinguishing feature of fungal abscesses [[6], [7], [8]].

DWI is particularly useful in detecting early ischemic changes in cerebral aspergillosis. It can demonstrate areas of restricted diffusion within the brain, corresponding to acute infarcts caused by vascular occlusion from fungal hyphae. These infarcts often precede the development of abscesses and are critical for early diagnosis. Gradient-echo sequences, on the other hand, are sensitive to hemorrhage and can detect even small amounts of blood within the brain parenchyma, which is common in aspergillosis due to the angioinvasive nature of the fungus [10,11]. In our patient, the imaging pattern, combined with the clinical context of a necrotic nasal lesion and positive fungal cultures, strongly suggested an IFI, leading to prompt antifungal therapy.

The differential diagnosis for multiple ring-enhancing lesions in an immunocompromised patient includes bacterial abscesses, metastatic disease, lymphoma, and mucormycosis. Mucormycosis is a critical differential in diabetic patients; however, *Aspergillus* abscesses often present with more distinct hemorrhagic borders and specific involvement of the basal ganglia and thalami [11].

The management of cerebral aspergillosis involves aggressive antifungal therapy, typically with agents such as voriconazole or liposomal amphotericin B, which have shown the best efficacy in penetrating the blood-brain barrier and combating fungal infections. Despite advancements in antifungal therapies, the prognosis remains poor, with mortality rates approaching 100% in many cases, particularly when diagnosis and treatment are delayed [5,7,12].

## Conclusion

Cerebral aspergillosis remains a challenging diagnosis with a poor prognosis, particularly in immunocompromised patients. Early recognition of characteristic imaging findings, combined with a high index of suspicion and prompt initiation of antifungal therapy, is critical for managing this life-threatening condition. While MRI and CT are the primary imaging modalities, the inclusion of advanced techniques such as DWI and gradient-echo sequences can provide additional diagnostic clarity and help guide treatment decisions.

## Patient consent

The informed written consent was obtained from the patient for publication of the case report and all imaging studies in RADIOLOGY CASE REPORTS.
